# Propofol induces MAPK/ERK cascade dependant expression of cFos and Egr-1 in rat hippocampal slices

**DOI:** 10.1186/1756-0500-3-201

**Published:** 2010-07-17

**Authors:** Srivatsan Kidambi, Joel Yarmush, Yevgeny Berdichevsky, Sangeetha Kamath, Wayne Fong, Joseph SchianodiCola

**Affiliations:** 1 Department of Anesthesiology, New York Methodist Hospital, Brooklyn, NY, USA; 2 The Center for Engineering in Medicine and Surgical Services, Massachusetts General Hospital, Shriners Hospitals for Children and Harvard Medical School, Boston, Massachusetts, USA; 3 Department of Chemical & Biomolecular Engineering, University of Nebraska, Lincoln, NE, USA

## Abstract

**Background:**

Propofol is a commonly used intravenous anesthetic agent, which produce rapid induction of and recovery from general anesthesia. Numerous clinical studies reported that propofol can potentially cause amnesia and memory loss in human subjects. The underlying mechanism for this memory loss is unclear but may potentially be related to the induction of memory-associated genes such as c-Fos and Egr-1 by propofol. This study explored the effects of propofol on c-Fos and Egr-1 expression in rat hippocampal slices.

**Findings:**

Hippocampal brain slices were exposed to varying concentrations of propofol at multiple time intervals. The transcription of the immediate early genes, c-Fos and Egr-1, was quantified using quantitative reverse transcriptase polymerase chain reaction (qRT-PCR). MAPK/ERK inhibitors were used to investigate the mechanism of action. We demonstrate that propofol induced the expression of c-Fos and Egr-1 within 30 and 60 min of exposure time. At 16.8 μM concentration, propofol induced a 110% increase in c-Fos transcription and 90% decrease in the transcription of Egr-1. However, at concentrations above 100 μM, propofol failed to induce expression of c-Fos but did completely inhibit the transcription of Egr-1. Propofol-induced c-Fos and Egr-1 transcription was abolished by inhibitors of RAS, RAF, MEK, ERK and p38-MAPK in the MAPK/ERK cascade.

**Conclusions:**

Our study shows that clinically relevant concentrations of propofol induce c-Fos and down regulated Egr-1 expression via an MAPK/ERK mediated pathway. We demonstrated that propofol induces a time and dose dependant transcription of IEGs c-Fos and Egr-1 in rat hippocampal slices. We further demonstrate for the first time that propofol induced IEG expression was mediated via a MAPK/ERK dependant pathway. These novel findings provide a new avenue to investigate transcription-dependant mechanisms and suggest a parallel pathway of action with an unclear role in the activity of general anesthetics.

## Introduction

Propofol is the most commonly used intravenous general anesthetic that has been proven to be highly effective due to its rapid onset and short recovery time after injection. Because of these advantages, propofol is now widely used both for general anesthesia and for sedation with local anesthesia[1,2]. It is thought to act primarily through the potentiation of γ-aminobutyric acid (GABA-A) receptor currents [3,4]. While the GABA-A dependant mechanisms is well established, there is a growing interest in elucidating secondary mechanisms that might have long-lasting side effects [5,6]. Propofol has been reported to produce amnesia in addition to sedation, hypnosis and general anesthesia. The inhibition of long-term potentiation (LTP) in the hippocampus has been attributed to the amnesic effect of propofol[3,7,8]. However, the underlying cellular mechanisms for the propofol inhibition of hippocampal LTP are poorly understood.

Recent studies have reported the important role played by the expression of rapidly inducible genes known as immediate-early genes (IEGs) in long-term potentiation (LTP) and memory consolidation [9]. Transcription factors, such as c-Fos, Egr-1, Nurr1 and Arc have been found to play a role in learning, memory and LTP [10,11]. Several commonly used anesthetic agents such as midazolam and thiopental were shown to elicit rapid and transient induction of several immediate early genes in neurons, including c-Fos, Egr-1 and Jun B [12,13]. However, the same studies demonstrated that high concentrations of propofol did not affect the expression of c-Fos, JunB or Egr-1 in culture [12,13]. In contrast, Kozinn and co-workers showed that propofol regulates the expression of c-Fos in hippocampal slices via inhibition of N-methyl-D-aspartate (NMDA) receptor activation of the extracellular signal-regulated kinase (ERK) pathway [14,15], while Hamaya et al reported that propofol increases the expression of c-Fos and Jun B in the rat brain [16]. Recently we have also demonstrated that propofol induces a time and dose dependant transcription of the IEGs c-Fos and Egr-1 in neuronal cells[17]. Therefore, the interaction between propofol and these immediate early transcription factors is still under debate.

In this study, we investigated the ability of propofol to induce the transcription of c-Fos and Egr-1 in rat hippocampal brain slices. Using this system we demonstrate a time and dose dependant transcription of c-Fos and Egr-1. Remarkably, while 16.8 μM of propofol, corresponding to plasma concentrations in general anesthesia, induced a 110% increase in c-Fos transcription, higher concentrations failed to induce any transcriptional changes in c-Fos. In contrast, propofol down regulated the expression of Egr-1 with increasing time and concentration. The changes in transcription of c-Fos and Egr-1 relied on the p38 mitogen-activated protein kinase (p38-MAPK)/ERK signaling cascade. These findings provide a new avenue to investigate transcription-dependant mechanisms and suggest a parallel pathway of action with an unclear role in the activity of general anesthetics.

## Methods

### Materials

All chemicals were purchased from Sigma (St Louis, MO) unless otherwise indicated. Propofol was purchased from AstraZeneca (Wilmington, DE). FTI-277 (RAS Inhibitor-Cat # 344555), RAF1 Kinase Inhibitor I (Cat # 553008), U0126 (MEK Inhibitor-Cat # 662005), PD98059 (ERK Inhibitor-Cat # 513000), and SB203580 (p38-MAPK Inhibitor-Cat # 559389), were purchased from Calbiochem (San Diego, CA).

### Hippocampal slices

Isolated hippocampi from postnatal day 7 Sprague-Dawley rat pups (Harlan Laboratories) were cut into 350 μm slices on a McIlwain tissue chopper (Mickle Lab Eng. Co., Surrey, UK). 15-17 hippocampal slices were prepared from each pup. Each data point in an experimental group represents one slice from one animal. Slices were placed into a 6-well plate that was filled with just enough artificial cerebrospinal solution (ACSF, composed of 120 mM NaCl, 3.3 mM KCl, 1.25 mM NaH_2_PO_4_, 26 mM NaHCO_3_, 1.3 mM CaCl_2_, 0.9 mM MgCl_2_, and 10 mM glucose in deionized water) to cover the bottom of each well. At this stage the brain slices were left untreated (negative control), treated with intralipid (vehicle control) or treated with increasing concentrations of propofol ranging from 5.6 to 112.2 μM propofol in ACSF and incubated in the interface configuration described above in a humidified 5% CO_2 _incubator at 37°C. The transcription of c-Fos and Egr-1 genes was measured at several time intervals post treatment. All animals were treated in accordance with National Research Council guidelines and approved by the Subcommittee on Research Animal Care at the Massachusetts General Hospital.

### Real-Time Quantitative Reverse Transcriptase

Analysis of c-Fos and Egr-1 transcription was carried out using Mx3000P QPCR system (Stratagene, La Jolla, CA). RNA from the cells was extracted and purified using a Qiagen's Nucleospin RNA II kit (Valencia, CA) and quantified using Nanodrop ND-1000 (Wilmington, DE). 100 ng of total mRNA was reverse transcribed to cDNA using a Superscript Platinum Two-Step qRT-PCR kit from Invitrogen Life Technologies (Carlsbad, CA) and amplified in a Perkin Etus Thermal Cycler 480. Reported values were normalized to the internal standard actin. The primer sequences were as follows: c-Fos, (forward) 5'-GAAGGAACCAGACAGGTCCA, (reverse) 5'-TCACCCTGCCTCTTCTCAAT (with expected product size of 381 bp); Egr1, (forward) 5'- AGCGAACAACCCTATGAGCA, (reverse) 5'-TCGTTTGGCTGGGATAACTC (with expected product size of 345 bp); Actin, (forward) 5'- GTCGTACCACTGGCATTGTG, (reverse) 5'-CTCTCAGCTGTGGTGGTGAA.

### Statistics

**Figure 1:**
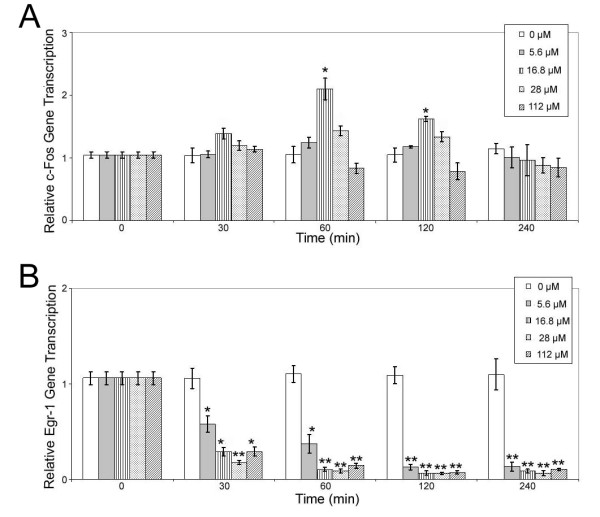
**Time and dose dependant propofol induced (A) c-Fos and (B) Egr-1 transcription in brain slices**. Transcription of c-Fos and Egr-1 was quantified using qRT-PCR. Gene transcription is normalized to actin and non-treated controls. Data are presented as mean ± sd from three independent experiments. * p < 0.05; ** p < 0.01 versus non-treated control.

**Figure 2:**
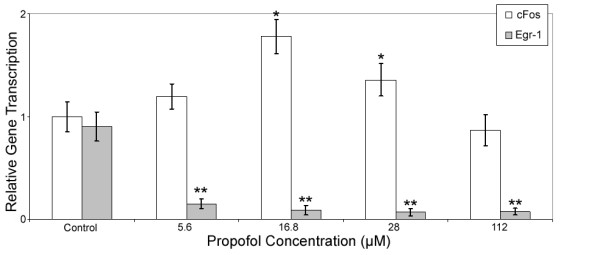
**Dose dependant expression of c-Fos and Egr-1 by propofol following 60 min stimulation**. The transcription of c-Fos and Egr-1 was quantified using qRT-PCR. Gene transcription is normalized to actin and to non-treated controls. Data are presented as mean ± sd from three independent experiments. * p < 0.05; ** p < 0.01 versus non-treated control.

**Figure 3:**
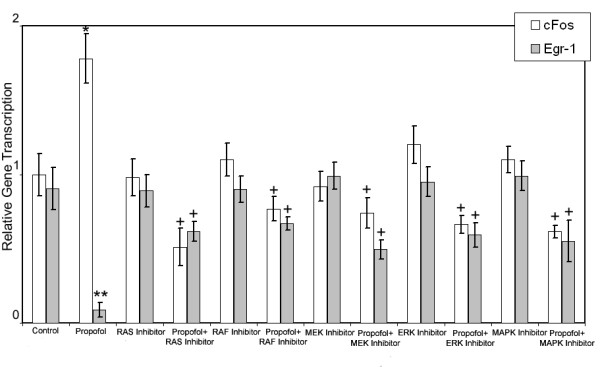
**Elucidating the mechanism of propofol-induced c-Fos and Egr-1 expression in hippocampus brain slices**. The transcription of c-Fos and Egr-1 was assessed using qRT-PCR. Brain slices with or without pretreatment of 10 μM FTI-277 (RAS Inhibitor), 10 μM RAF1 Kinase Inhibitor I, 10 μM UO126 (MEK Inhibitor), 50 μM PD98059 (ERK Inhibitor), and 10 μM SB203580 (p38-MAPK Inhibitor), were stimulated with 16.8 μM propofol for 60 min and the expression of c-Fos and Egr-1 was measured using qRT-PCR. Data are presented as mean ± sd from three independent experiments. * p < 0.05 versus control, + p < 0.05 versus propofol treatment.

Data were analyzed using two-way ANOVA (for Figure 1) and the Student's *t*-test (for Figures 2 and 3). Results are presented as means ± standard deviation and P values < 0.05 were considered statistically significant.

## Results

To investigate the effect of propofol on the transcription of c-Fos and Egr-1, we exposed the hippocampal slices to varying concentrations of propofol at increasing time intervals. The transcription of c-Fos (Figure 1A) peaked between 30 and 60 min of exposure and rapidly returned to control levels while the transcription of Egr-1 (Figure 1B) decreased and reached the minimum at 240 min. Maximal induction of c-Fos occurred following 60 min stimulation with 16.8 μM propofol, resulting in 110% ± 17% (P = 0.012, N = 3) increase in transcription. Surprisingly, both lower and higher doses of propofol resulted in a non-significant, but sub-maximal induction of c-Fos transcription 24% ± 9% (P = 0.073, N = 3) and 43% ± 7% (P = 0.065, N = 3) increase for 5.6 μM and 28.0 μM concentration respectively. In contrast, propofol caused a time and concentration dependant down regulation of Egr-1 transcription and was completely inhibited at 60 min and remained inhibited for up to 240 min for all concentrations. Clinically relevant concentration of 16.8 μM propofol resulted in 91% ± 4% fold (P = 0.006, N = 3) inhibition in Egr-1 transcription. Both lower and higher doses of propofol resulted in significant decrease of Egr-1 transcription, 87% ± 5% (P = 0.032, N = 3) and 94% ± 3% (P = 0.008, N = 3) decrease for 5.6 μM and 28.0 μM concentration respectively.

To further probe the dose-dependence of c-Fos and Egr-1 transcription on propofol concentration, we exposed the hippocampal slices to increasing concentrations of propofol for 60 min. Figure 2 shows that at clinically relevant concentrations (16.8 μM) propofol caused a 80% induction in c-Fos transcription, and a 91% decrease in the transcription of Egr-1. However, at a dose of 112.2 μM, propofol did not significantly alter c-Fos (13% ± 1.5% decrease; P = 0.188, N = 3) compared to control. In contrast, propofol significantly reduced the transcription of Egr-1 in all concentrations including both lower concentration of 5.6 μM (85% ± 5% decrease; P = 0.008, N = 3) and higher concentration of 112.2 μM (93% ± 3% decrease; P = 0.007, N = 3). Further, clinically relevant concentration of 16.8 μM propofol resulted in 91% ± 4% (P = 0.006, N = 3) decrease in Egr-1 transcription.

To evaluate the role of the MAPK/ERK signaling cascade in the induction of c-Fos and Egr-1 expression we exposed hippocampal slices pretreated with 10 μM FTI-277 (RAS Inhibitor), 10 μM RAF1 Kinase Inhibitor I, 10 μM UO126 (MEK Inhibitor), 50 μM PD98059 (ERK Inhibitor), or 10 μM SB203580 (p38-MAPK Inhibitor) to propofol (Figure 3; Table 1). Pretreatment of hippocampal slices with RAS, RAF, MEK, ERK or p38-MAPK inhibitors for 60 min did not significantly change the transcription of c-Fos or Egr-1. Stimulation of RAS inhibitor pretreated hippocampal slices with 16.8 μM propofol resulted in a significantly lower c-Fos (49% ± 12%; P = 0.024, N = 3) and higher Egr-1 (39% ± 7%; P = 0.046, N = 3) gene transcription compared to propofol treatment alone. Similarly, stimulation of hippocampal slices pretreated with RAF, MEK, ERK or p38-MAPK inhibitors with 16.8 μM of propofol resulted in a significantly lower c-Fos and higher Egr-1 gene transcription compared to propofol treatment alone, demonstrating the role of p38-MAPK/ERK signaling cascade in the propofol induction of the IEGs, c-Fos and Egr-1.

**Table 1:** Gene expression of c-Fos and Egr-1 accessed using RT-PCR

Conditions	c-Fos		Egr-1	
	
	Fold change	P value	Fold change	P value
**Propofol**	1.78 ± 0.17		0.09 ± 0.04	

**Propofol + RAS inhibitor**	0.51 ± 0.12	0.024	0.62 ± 0.09	0.046

**Propofol + RAF inhibitor**	0.77 ± 0.08	0.041	0.67 ± 0.14	0.032

**Propofol + MEK inhibitor**	0.74 ± 0.10	0.036	0.49 ± 0.09	0.029

**Propofol + ERK inhibitor**	0.66 ± 0.11	0.032	0.59 ± 0.08	0.044

**Propofol + MAPK inhibitor**	0.62 ± 0.13	0.021	0.55 ± 0.14	0.037

## Discussion

The purpose of this study was to evaluate the effects of propofol stimulation on c-Fos and Egr-1 gene transcription in hippocampal brain slices at varying doses and time intervals. Our data also suggests that the induction of c-Fos and Egr-1 by propofol is mediated by the p38-MAPK/ERK signaling cascade.

The data shows a time and dose dependant induction of c-Fos and Egr-1 expression in hippocampal brain slices following exposure to propofol. These results are especially interesting as previous reports suggested that propofol did not affect the expression of c-Fos and Egr-1 [12-15]. Kozinn and co-workers demonstrated that lower concentrations of propofol inhibits N-methyl-D-aspartate (NMDA) receptor activation of MAPK/ERK pathway and c-Fos transcription in hippocampal neurons[14]. However, our work demonstrates that propofol has a significant effect at higher concentrations and when exposed to longer time. In fact, in a clinical setting, propofol concentration in the plasma is about 16.8 μM [18], and at this concentration the drug induced a 110% increased expression of c-Fos and 90% decrease of Egr-1 transcription. Jevtovic-Todorovic and co-workers demonstrated that exposure of the developing brain to drugs that block NMDA glutamate receptors or drugs that potentiate GABA(A) receptors can induce apoptotic neurodegeneration in the developing brain, deficits in hippocampal synaptic function, and persistent memory/learning impairments[19]. Our results also suggest that clinically relevant doses of propofol can potentially induce long-term changes in neuronal function by inducing changes in the gene expression.

Mitogen-activated protein kinases (MAPKs), a large family of cytosolic and nuclear serine/threonine kinases, have been implicated in the regulation of variety of cellular and synaptic activities in neurons[20]. Specifically, MAPKs form central signaling pathways processing inducible gene expression in response to various forms of extracellular and intracellular stimuli. The activation of MAPK/ERK signaling pathway has been demonstrated to be a critical molecular step toward the development and/or maintenance of synaptic plasticity including LTP and long-term depression (LTD) and memory formation in the hippocampus[21-23]. Several studies have shown that general anesthetics may interfere with cellular targets, including MAPK/ERK pathways [14,24]. Previous reports suggest the involvement of the MAPK/ERK pathway in the induction of c-Fos and Egr-1 expression by growth hormone[25] or midazolam[12].

Our results demonstrate that inhibition of the MAPK/ERK signaling cascade using RAS, RAF, MEK, ERK or p38-MAPK inhibitors blocked the propofol-induced c-Fos and Egr-1 expression, thus suggesting the possible involvement of MAPK/ERK pathway in propofol-induced immediate early gene expression. These findings indicate a new avenue to explore a transcription-dependant mechanism that may possibly underlie anesthetic interference with synaptic plasticity related to amnesic properties of intravenous anesthetics.

## Conclusion

In summary, we demonstrated that propofol induces a time and dose dependant transcription of IEGs c-Fos and Egr-1 in rat hippocampal slices. We further demonstrate for the first time that propofol induced IEG expression was mediated via a MAPK/ERK dependant pathway.

## Competing interests

The authors declare that they have no competing interests.

## Authors' contributions

SK conceptualized the study, designed the study protocols and analyzed the data; SK, JY, YB, SKam, WF, and JS performed research; SK, JY and JS contributed new reagents/analytic tools; and SK drafted the manuscript. All authors approved the final version of the manuscript.
